# Pathways between Primary Production and Fisheries Yields of Large Marine Ecosystems

**DOI:** 10.1371/journal.pone.0028945

**Published:** 2012-01-20

**Authors:** Kevin D. Friedland, Charles Stock, Kenneth F. Drinkwater, Jason S. Link, Robert T. Leaf, Burton V. Shank, Julie M. Rose, Cynthia H. Pilskaln, Michael J. Fogarty

**Affiliations:** 1 National Marine Fisheries Service, Narragansett, Rhode Island, United States of America; 2 NOAA Geophysical Fluid Dynamics Laboratory, Princeton, New Jersey, United States of America; 3 Institute of Marine Research and Bjerknes Centre for Climate Research, Bergen, Norway; 4 National Marine Fisheries Service, Woods Hole, Massachusetts, United States of America; 5 National Marine Fisheries Service, Milford, Connecticut, United States of America; 6 School for Marine Science and Technology, New Bedford, Massachusetts, United States of America; Aristotle University of Thessaloniki, Greece

## Abstract

The shift in marine resource management from a compartmentalized approach of dealing with resources on a species basis to an approach based on management of spatially defined ecosystems requires an accurate accounting of energy flow. The flow of energy from primary production through the food web will ultimately limit upper trophic-level fishery yields. In this work, we examine the relationship between yield and several metrics including net primary production, chlorophyll concentration, particle-export ratio, and the ratio of secondary to primary production. We also evaluate the relationship between yield and two additional rate measures that describe the export of energy from the pelagic food web, particle export flux and mesozooplankton productivity. We found primary production is a poor predictor of global fishery yields for a sample of 52 large marine ecosystems. However, chlorophyll concentration, particle-export ratio, and the ratio of secondary to primary production were positively associated with yields. The latter two measures provide greater mechanistic insight into factors controlling fishery production than chlorophyll concentration alone. Particle export flux and mesozooplankton productivity were also significantly related to yield on a global basis. Collectively, our analyses suggest that factors related to the export of energy from pelagic food webs are critical to defining patterns of fishery yields. Such trophic patterns are associated with temperature and latitude and hence greater yields are associated with colder, high latitude ecosystems.

## Introduction

A central principle of an ecosystem-based approach to fisheries management is the recognition that fishery yields are ultimately limited by ecosystem primary production [1], [2]. Because of the necessity to predict future fishery yields, the environmental mechanisms that determine the magnitude of ecosystem-level production has received a great deal of attention [3]. Though the “bottom up” model to describe the productivity of fishery resources has been tested in a variety of ways and across a range of ecosystem types including coastal lagoons, estuaries, open marine systems, and freshwater environments [4], [5], [6], much ambiguity remains regarding the predictive value of metrics of primary productivity to estimate fishery production. Freshwater ecosystems, which are characterized by well defined boundaries, tractable conduits of energy flows, and naturally occurring spatial and temporal variations in nutrient loadings provide *de facto* experimental units of observation and enable the quantification of productivity at different trophic levels [7], [8], [9]. Anthropogenic impacts such as increased loadings of phosphorus in freshwater ecosystems have been associated with increased phytoplankton biomass and subsequent fish yields in many lake ecosystems [10]. In contrast, estimating the yield potential in marine ecosystem is a greater challenge [11]. Most marine systems have extensive open boundaries and thus limited constraints on the exchange of nutrients, organic material, and migratory fauna with neighboring waters. Additionally, the large spatial scales of marine ecosystems complicate estimates of yield potential due to inadequacies in sampling. Nonetheless, both regional and global efforts have been made.

Regional-scale analyses, those that have analyzed a subset of world ocean basins or some portion of a basin, have used both measured and proxy estimates of primary production as a predictor of the associated fish yield. One often-used proxy of primary production is chlorophyll *a* concentration. Resident fish yield in the Northeast Pacific for eleven different fishing areas was strongly and linearly correlated (r^2^ = 0.87) with the mean chlorophyll *a* concentration, which provided an adequate proxy of primary production in this system [12]. Similarly, a strong linear correlation between chlorophyll *a* and fisheries yield (r^2^ = 0.69) was found across nine coastal areas in the Northwest Atlantic [13]. Both of these studies elected to use chlorophyll concentration as a proxy for primary production under the assumption that the two scaled linearly. Similar results were also obtained with estimated rates of primary production as the independent variable. A survey of both North Atlantic and North Pacific ecosystems yielded a linear relationship between annual fish catch and primary production [14]. In an analysis of fourteen eco-regions of Northeast Atlantic seas, strong relationships between primary production and yields were reported both for plankton feeders (r^2^ = 0.73) and for all fish species (r^2^ = 0.64) [15]. Iverson [6] found a significant linear relationship between carnivorous fish and squid production and new primary production across open ocean and coastal environments (r^2^ = 0.96), but the analysis included only 10 sites and excluded upwelling regions and areas with strong tidal mixing. In the regional analyses for marine ecosystems, where yield and some measure of primary production have been examined, the linear relationship is generally positive; however, this relationship is not consistent when global patterns are examined.

Robust relationships between estimates of primary production and fisheries yield at global scales have been difficult to discern. In an analysis of global fishery yields disaggregated by 64 globally-distributed Large Marine Ecosystems (LMEs), total yields were found to scale with rates of net primary production (NPP, C m^−2^ y^−1^) [16]. However, the interpretation of this relationship is problematic since yields were not scaled by LME areas, thus only NPP was characterized on an areal basis. A similar analysis was performed by Chassot et al. [17] with both LME yield and NPP characterized on an areal basis. Quantile regressions (50%, 90%) of mean annual catch determined by satellite-estimated primary production resulted in positive relationships when the regressions were forced through the origin. However, qualitative comparison of these models relative to regional relationships, suggests that the effectiveness of primary production as a predictor of catch declines significantly for the global scale analysis.

The ineffectiveness of primary production as an indicator of fisheries yield at a global scale is consistent with theoretical arguments supporting a more nuanced and complex relationship between the two quantities. Ryther [18] for example, argues that shifts in the size structure of the phytoplankton community to larger phytoplankton and increasing consumer gross growth efficiencies in more productive ecosystems should result in greater fisheries production per unit of primary production. The importance of particle export fluxes, or the fraction of primary production exported from pelagic foodwebs via sinking particles, can vary in complex ways with planktonic foodweb, water column structure, temperature, and ecosystem disturbance [19], [20], [21], [22], [23], [24]. These flux rates can also strongly influence trophic transfer within ecosystems [25], [26], [27]. In this paper, we assess primary productivity and a collection of additional variables as predictors of fisheries yields for 52 of the 64 of the globally-distributed Large Marine Ecosystems. The additional variables include simple geographic, physical, and biological variables that are readily observed (latitude, temperature, chlorophyll concentration) as well as derived quantities which may more accurately indicate differences in the export of energy from the planktonic ecosystem to fisheries across ecosystems on a global scale (e.g., particle export fluxes and estimated mesozooplankton production).

## Materials and Methods

### Fishery Yields of Large Marine Ecosystems

Fishery yields for all landed species were obtained from the Sea Around Us project dataset [28]. The geographic distribution of yields can be parsed in spatially a number of ways; we utilized the LME convention that divides the continental shelves and inland seas of the world into 64 ecosystems [16]. The fishery yield data are spatially explicit and are based on the Food and Agriculture Organization (FAO) of the United Nations catch statistics and other similar sources of information for the period 1950–2006 [29]. These data have been corrected for illegal and unreported catches, which in some areas may be substantial [30]. Following Chassot et al. [17], fishery yield data for eight of the LMEs were excluded from the analysis: Antarctic, Arctic Ocean, Beaufort Sea, Chukchi Sea, East China Sea, East Siberian Sea, Hudson Bay, Kara Sea, Laptev Sea, and Yellow Sea. The landings data for these ecosystems were considered problematic for a number of reasons including inherently unreliable data and the effect of ice conditions on fishery operations. We also excluded the Baltic and Black Sea LMEs because in many ways these inland sea ecosystems are not readily comparable to continental shelf marine ecosystems.

We computed four landings summaries: total catches of all species; landings of functional groups considered to be pelagic feeders (see Table 1 for proportion of functional groups allocated to pelagic feeding where some groups were partitioned to half pelagic and half demersal feeding), landings of functional groups considered to be demersal feeders or the balance of landings not allocated to pelagic landings; and, landings of functional groups considered to be resident or non-migratory which included all landings except medium and large pelagic landings [17]. These were expressed as monthly yields per square kilometer of the LME for ice free months by dividing the landings summary by the LME area and by an estimated number of ice free months from satellite data. Sea ice concentration was extracted from the daily optimum interpolation sea surface temperature (OISSTv2) analysis database [31]. This analysis has a spatial resolution of 0.25°×0.25° and a temporal resolution of 1 day and is based on data from the Advanced Very High Resolution Radiometer (AVHRR) infrared satellite. Monthly sea ice concentration, expressed as a percentage, was extracted for the years 1982–2009 and averaged for each LME. For each LME yield time series, we considered time series mean, median, third quartile and maximum as proxy quantities to represent the sustainable yield of the LME. Within each summary, these measures were highly correlated (no correlation coefficient between time series statistics was less than 0.96), suggesting that any of the four statistics could be used equivalently in comparing yields to independent variables. We selected the third quartile statistic as representative of sustainable yield levels.

**Table 1:** Weights (proportion attributed to pelagic feeding) applied to functional groups of fishery landings.

Functional Groups	Weight
Cephalopods	0.5
Krill	1
Large bathydemersals (> = 90 cm)	0.5
Large benthopelagics (> = 90 cm)	1
Large demersals (> = 90 cm)	0
Large flatfishes (> = 90 cm)	0
Large pelagics (> = 90 cm)	1
Large reef assoc. fish (> = 90 cm)	0
Large sharks (> = 90 cm)	0.5
Lobsters, crabs	0
Medium bathydemersals (30–89 cm)	0
Medium bathypelagics (30–89 cm)	1
Medium benthopelagics (30–89 cm)	0.5
Medium demersals (30–89 cm)	0
Medium pelagics (30–89 cm)	1
Medium reef assoc. fish (30–89 cm)	0
Other demersal invertebrates	0
Other groups	0.5
Shrimps	0.5
Small bathydemersals (<30 cm)	0
Small bathypelagics (<30 cm)	0
Small benthopelagics (<30 cm)	0.5
Small demersals (<30 cm)	0
Small pelagics (<30 cm)	1
Small reef assoc. fish (<30 cm)	0.5
Small to medium flatfishes (<90 cm)	0
Small to medium rays (<90 cm)	0.5
Small to medium sharks (<90 cm)	0.5

The relationship between fisheries yields and each predictor variable was quantified using both Spearman rank order correlation and Pearson product-moment correlation. The significance of the Spearmen rank order correlation can be assessed for data with any underlying distribution and was thus calculated using untransformed variables. The significance test of the Pearson product-moment correlation requires that the data be distributed bivariate normal [32]. The distribution of monthly yields and other variables used in the analysis were thus tested for normality with the Shapiro-Wilk *W* statistic and with the inspection of frequency distribution and normal probability plots. If a variable was deemed non-normal, it was transformed with four candidate transforms including log, square root, cube root and fourth root. The transform providing the most improvement of the *W* statistics was applied to the data. Fourth root transforms was the most appropriate transformation for the yield data.

### Sea Surface Temperature and Latitude of Large Marine Ecosystems

Sea surface temperature was extracted from the OISSTv2 analysis, the same dataset used for the sea ice data [31]. SST was expressed as an annual mean for each LME for the period 1982–2009. LME latitude was the latitude of the LME centroid expressed as absolute values in our analysis. No transformation was required for the SST data for the Pearson product-moment correlation analysis whereas the latitude data was transformed with a square root transform.

### Chlorophyll *a* Concentration in Large Marine Ecosystems

Chlorophyll *a* concentrations were derived from satellite remote sensing data collected from the Sea-viewing Wide Field of View Sensor (SeaWiFS) sensor. We used the monthly level-3 processed data averaged over each LME for the period 1998–2009 to compute an annual mean concentration (mg m^−3^) (http://oceancolor.gsfc.nasa.gov). Chlorophyll concentrations were fourth root transformed for the Pearson product-moment correlation analysis.

### Net Primary Production in Large Marine Ecosystems

There are a large number of algorithms used to estimate NPP from remote sensing data. The remote sensing community has conducted a series of reviews titled “primary production algorithm round robins” (PPARR) that evaluated NPP models using a range of performance statistics and differing calibration datasets [33], [34], [35], [36]. We focused our attention on two model formulations included in the PPARR evaluations that are provided to the scientific community via the Ocean Productivity website at Oregon State University (http://www.science.oregonstate.edu/ocean.productivity). The Vertically Generalized Production Model (VGPM) estimates NPP using photosynthetically available radiation, chlorophyll, an estimate of the euphotic zone depth, and a temperature-dependent estimate of the maximum photosynthetic rate [37]. This algorithm uses a 6^th^ order polynomial to describe the relationship between the maximum photosynthetic rate and temperature which was derived from a North Atlantic dataset and exhibits a peak near 20°C. The *Eppley*-VGPM replaces the 6^th^-order polynomial with a monotonically increasing exponential relationship supported by global compilations of phytoplankton growth rate data [38], [39].

The difference in the temperature dependencies used in the VGPM and Eppley-VGPM models is a primary cause of uncertainty in satellite-based primary production algorithms [35]. However, in the most recent round of PPARR comparisons [33], the *Eppley*-VGPM had root mean square difference (RMSD) errors that were lower than the VGPM model in 9 of 10 study sites (see Fig. 3 from [33]) and was among the group of highest ranked models in 7 of the 10 sites. The VGPM model, in contrast, was amongst the best models in only 3 sites. This difference in model performance with global datasets highlights the concern that the original formulation of the VGPM model is limited by the geographic range of the data used to tune the model [37] and should not be expected to provide robust results on a global scale.

**Figure 1:**
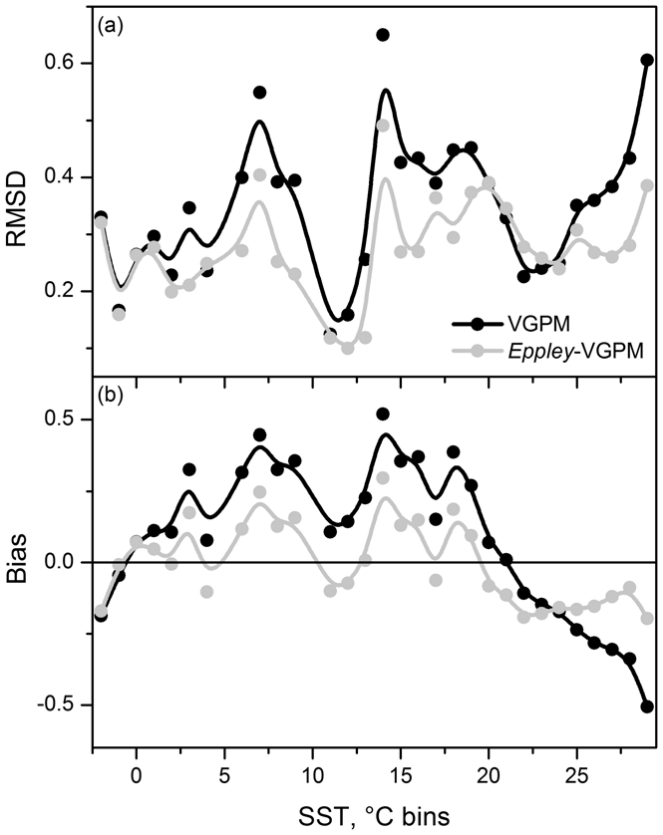
RMSD error and bias for two primary production algorithms. (a) RMSD for VGPM and *Eppley*-VGPM estimates of NPP based on the observational database of NPP from the fourth PPAR comparison binned by SST. (b) Bias associated with VGPM and *Eppley*-VGPM estimates of NPP binned by SST.

We performed an additional comparison to confirm that the *Eppley*-VGPM model was a robust model upon which to base globally-distributed primary production estimates for our study. Using the methods and supplementary data from Saba et al. [33], we computed RMSD and bias for VGPM and *Eppley*-VGPM models, but instead of evaluating the data by study site, we evaluated the data by temperature at the sample collection site. RMSD error was greater for VGPM model estimates over most of the temperature range of the PPARR dataset (Fig. 1a). Furthermore, VGPM model estimates tended to be biased high in low temperature water and biased low in high temperature water (Fig. 1b). The *Eppley*-VGPM estimates had a more balanced pattern of biases over the SST range. We thus used the monthly *Eppley*-VGPM NPP data based on chlorophyll concentrations from the SeaWiFS sensor in our analysis. *Eppley*-VGPM NPP was transformed with a log transform for the Pearson product moment correlation analysis.

### Particle Export Ratio and Flux in Large Marine Ecosystems

**Figure 2:**
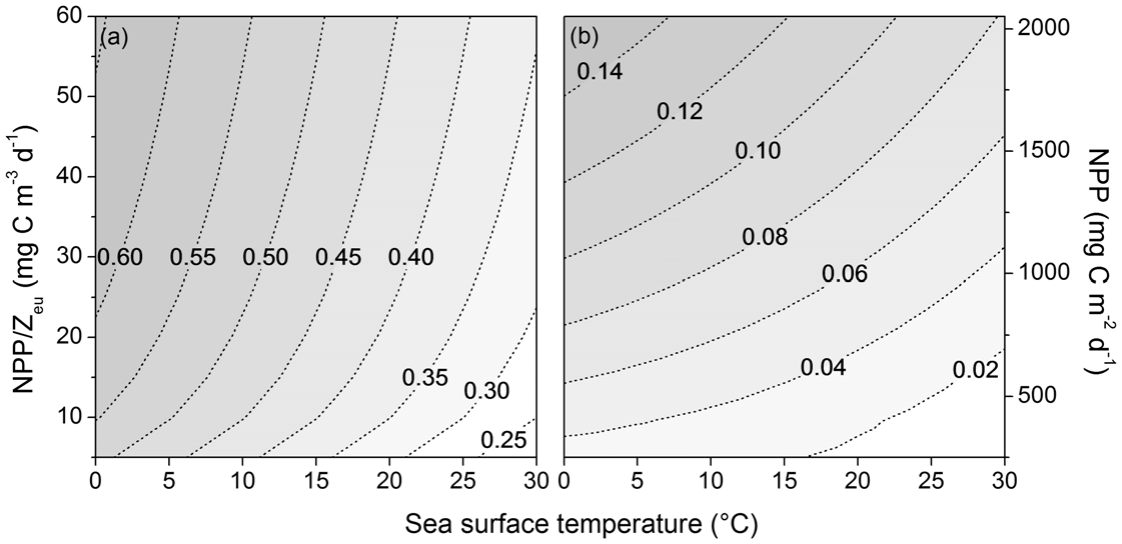
Contours of *pe*-ratio and *z*-ratio. (a) Contours of *pe*-ratio and (b) *z*-ratio as functions of SST and NPP/Z_eu_ and NPP, respectively.

There are a number of published models to estimate the ratio between rapidly sinking particulate matter from the euphotic zone and primary production, or the particle export ratio (*pe*-ratio, see review in Dunne et al. [22]). This quantity is closely related to the *f-*ratio which characterizes new vs. recycled production [19]. Observed *pe*-ratio trends include decreasing ratios with increasing temperature (often attributed to more rapid remineralization of particulate material), decreasing *pe*-ratios with increasing euphotic zone depth (attributed to extended time in the euphotic zone before export), and increasing *pe*-ratios with increasing primary productivity (often attributed to a shift toward larger plankton that produce more sinking material as productivity increases). We used the multi-linear regression fit provided in Dunne et al. [22] to estimate *pe*-ratio, which is given as:
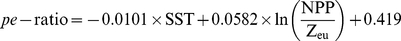
Where Z_eu_ is the euphotic zone depth, estimated from surface chlorophyll using the relationships of Morel and Berthon [40].
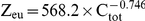
The variable C_tot_ is the total pigment or total chlorophyll *a* content within the euphotic layer, which is scaled non-linearly with chlorophyll concentration in the surface layer of the ocean, C_sur_.
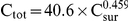
The relationship in Fig. 2a shows the range of parameter space encompassed by the LMEs considered herein. The *pe*-ratio based on *Eppley*-VGPM was log-normally distributed so a log-transformed was used for the Pearson product-moment correlation analysis. The product of *pe*-ratio and NPP yields an estimate of particle export flux, *pe*-flux, which unlike the scalar of *pe*-ratio represents a system flux rate. This flux was log transformed for the Pearson product-moment correlation analysis.

### *z*-ratio and Mesozooplankton Productivity in Large Marine Ecosystems

We estimated the ratio of mesozooplankton productivity to primary productivity (*z*-ratio) for each LME from the model estimates provided in Stock and Dunne [41]. This model was calibrated against 72 *z*-ratio estimates derived from *in situ* measurements of primary production, mesozooplankton biomass, and empirically-derived estimates of mesozooplankton growth rates [42] taken from a broad range of globally distributed ecosystems. It was then evaluated for 6,000 *z*-ratio estimates obtained from the mesozooplankton biomass estimates from the COPEPOD database [43], satellite-based primary production estimates, and empirical estimates of mesozooplankton growth. The model captures a modest positive and statistically significant (r = 0.4, p = <0.01) large-scale trend in *z*-ratios, but is characterized by considerable small-scale variability from fluctuations in mesozooplankton biomass. The model indicates a pronounced increase in the *z*-ratio as primary productivity increases, with the transition to high *z*-ratios occurring at lower NPP in cold water (Fig. 2b). The mechanisms underlying these patterns are the same as those identified by Ryther [18]: 1) a shift toward primary production by large phytoplankton as primary productivity increases, and 2) an increase in zooplankton growth efficiencies with increasing primary production as ingestion rates become large relative to basal metabolic costs.

The *z*-ratio estimates for each LME were derived from Fig. 2b and transformed with a square root transform for the Pearson product-moment correlation analysis. The product of *z*-ratio and NPP yields an estimate of mesozooplankton productivity, which unlike the scalar of *z*-ratio represents a system flux rate. This productivity rate was log transformed for the Pearson product-moment correlation analysis.

### Multivariate Analysis

We examined the total capacity of the independent variables to predict fishery yields using Partial Least Squares Regression (PLSR) [44]. We used all the variables, transformed and scaled to unit variance, to model each of the fishery yield groups and examined the first two principal components. We determined the contributions of the independent variables to each principal component by calculating the Pearson correlation between independent variables and the predicted values for the component.

## Results

### Association Between Sea Surface Temperature and Latitude and Yield

**Figure 3:**
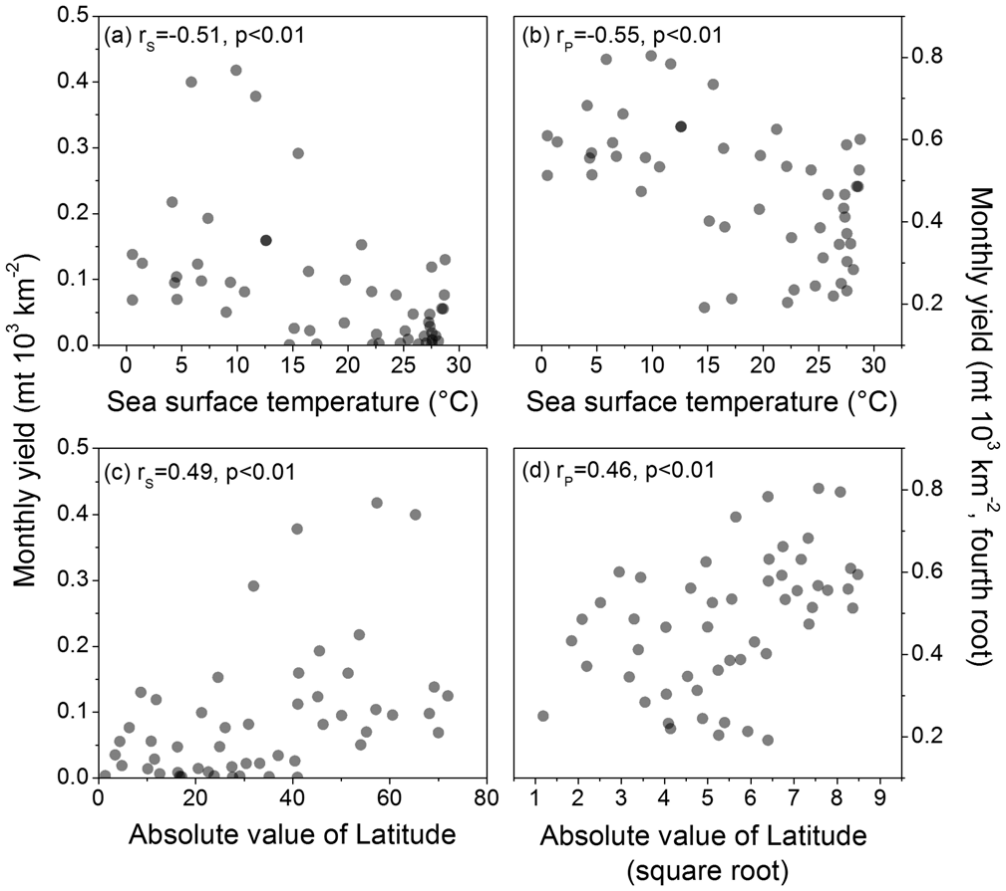
Fishery yield versus ecosystem temperature and absolute value of latitude. Scattergrams of: (a) total monthly fishery yield and mean annual sea surface temperature; (b) same data as panel (a) transformed to approximate bivariate normality; (c) total monthly fishery yield and absolute value of latitude; and (d) same data as panel (c) transformed to approximate bivariate normality. N = 52, the total number of LMEs in the analysis, for all plots. r_S_ = Spearman rank-order correlation and r_P_ = Pearson product-moment correlation.

Total monthly fisheries yield is correlated to annual mean SST and LME centroid latitude in a complementary fashion: greater yield is associated with lower mean SST and positively correlated with higher latitudes (Figs. 3a&b). The primary effect of the transformation is to accentuate the variability in the low fisheries-yield ecosystems (Figs. 3c&d), while the untransformed analysis is more strongly influenced by variability amongst the high-yield systems. The correlation coefficients associated with all the yield summaries were significant; the coefficients for total, pelagic and resident yield were of greater magnitude than the coefficient associated with the demersal yield (Table 2).

**Table 2:** Spearman rank-order and Pearson product-moment correlation between fisheries yields and factors for untransformed and transformed data, respectively.

	Monthly Yield			
Factor	Total	Pelagic	Demersal	Resident
Untransformed data, Spearman Rank-order Correlation:				
Sea Surface Temperature	−0.51**	−0.58**	−0.38**	−0.56**
|Latitude|	0.49**	0.54**	0.41**	0.53**
Chlorophyll *a*	0.68**	0.69**	0.59**	0.70**
Net primary production	0.06	0.01	0.09	0.01
*pe*-ratio	0.69**	0.72**	0.60**	0.74**
*pe*-flux	0.38**	0.33**	0.36**	0.35*
*z*-ratio	0.59**	0.58**	0.51**	0.58**
Mesozooplankton productivity	0.33*	0.28*	0.33*	0.31*
Transformed data, Pearson Product-moment Correlation:				
Sea Surface Temperature	−0.55**	−0.58**	−0.42**	−0.60**
|Latitude|	0.46**	0.49**	0.35*	0.50**
Chlorophyll *a*	0.70**	0.68**	0.64**	0.70**
Net primary production	0.05	−0.02	0.13	−0.01
*pe*-ratio	0.70**	0.71**	0.59**	0.73**
*pe*-flux	0.39**	0.33**	0.42**	0.36**
*z*-ratio	0.57**	0.51**	0.55**	0.55**
Mesozooplankton productivity	0.33*	0.27	0.37**	0.29*

Significant correlations are in bold type, with associated probabilities indicated with “*” for p = 0.05 and “**” for p = 0.01. N = 52 for all correlation coefficients.

### Association Between Chlorophyll Concentration and Yield

**Figure 4:**
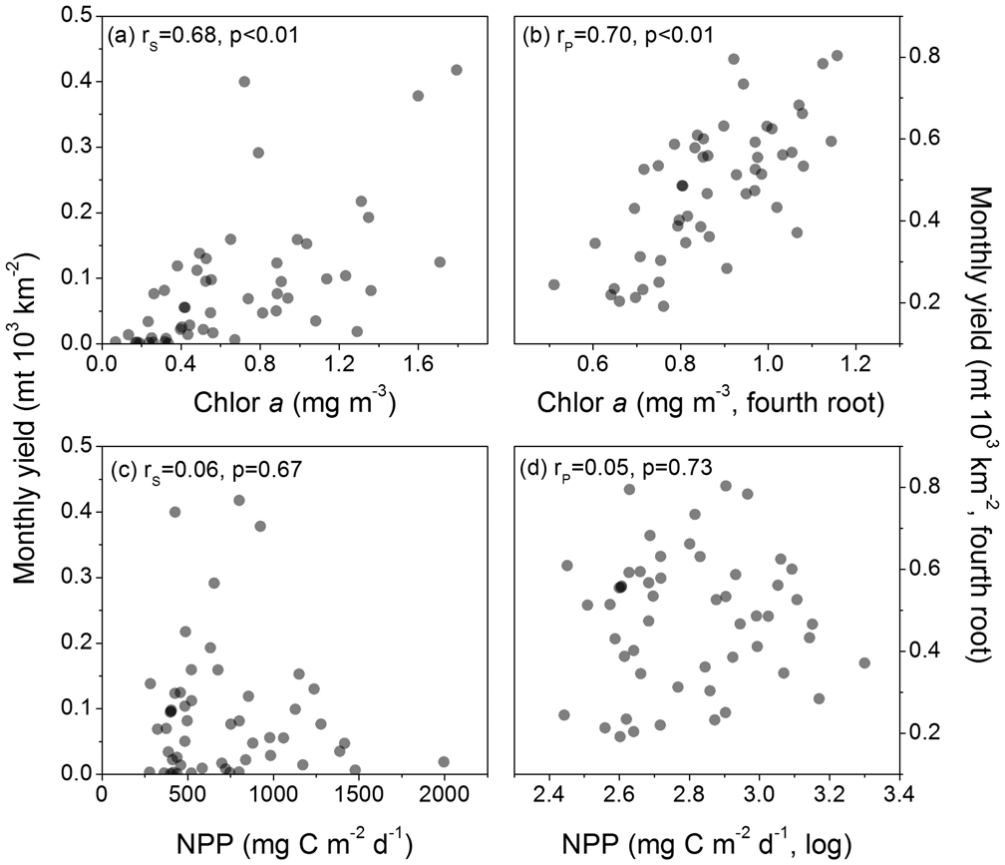
Fishery yield versus chlorophyll concentration and net primary production. Scattergrams of: (a) total monthly fishery yield and mean annual chlorophyll concentration; (b) same data as panel (a) transformed to approximate bivariate normality; (c) total monthly fishery yield and NPP computed using the *Eppley*-VGPM algorithm; and (d) same data as panel (c) transformed to approximate bivariate normality. N = 52, the total number of LMEs in the analysis, for all plots. r_S_ = Spearman rank-order correlation and r_P_ = Pearson product-moment correlation.

Total monthly fisheries yield was positively and significantly correlated (p<0.01) with chlorophyll *a* concentration (Figs. 4a&b) for both the untransformed and transformed data (Table 2). All the correlation coefficients associated with the individual functional group yield summaries were significant; the coefficients associated with the demersal yield were slightly lower than the other summaries (Table 2).

### Association Between Net Primary Production and Yield

The *Eppley*-VGPM NPP resulted in a bivariate distribution with respect to total fisheries yield with no detectable correlation using either the untransformed or transformed data (Figs. 4c&d). All the correlation coefficients associated with yield and NPP data were non-significant with some tending towards negative sign (Table 2).

### Association Between Particle Export Ratio and Flux and Yield

**Figure 5:**
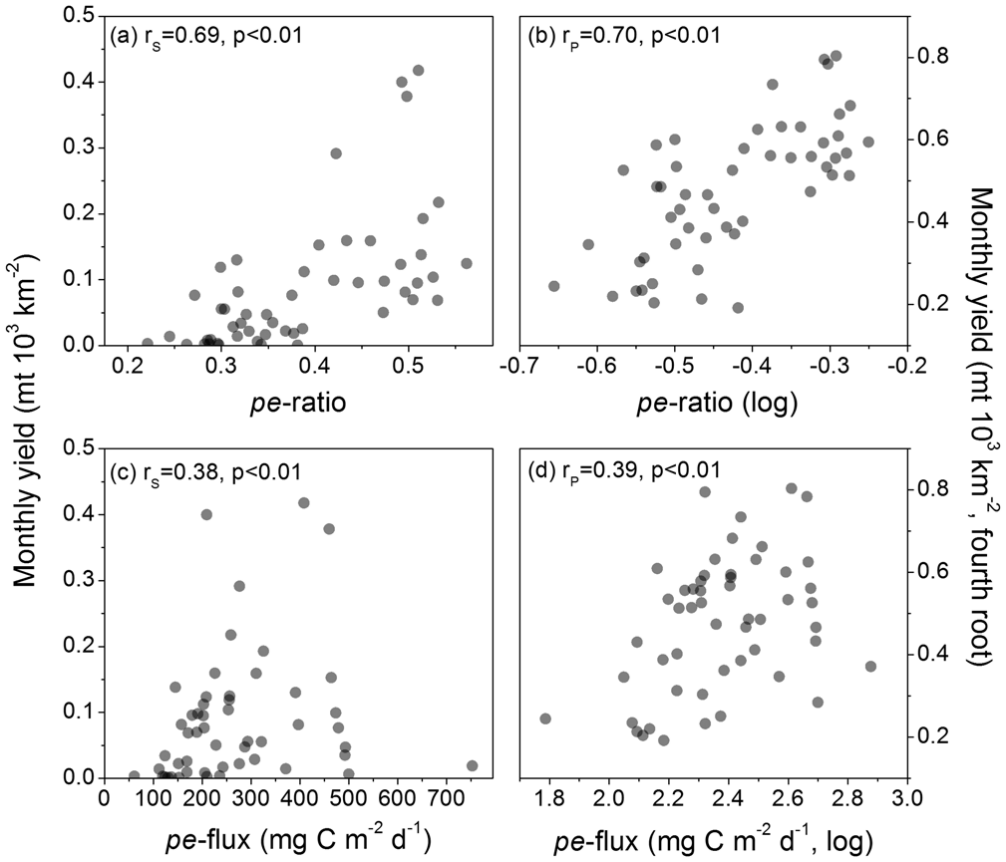
Fishery yield versus particle export ratio and particle export flux. Scattergrams of: (a) total monthly fishery yield and *pe*-ratio; (b) same data as panel (a) transformed to approximate bivariate normality; (c) total monthly fishery yield and *pe*-flux computed using the *Eppley*-VGPM algorithm; and (d) same data as panel (c) transformed to approximate bivariate normality. N = 52, the total number of LMEs in the analysis, for all plots. r_S_ = Spearman rank-order correlation and r_P_ = Pearson product-moment correlation.

Monthly fisheries yield was highly correlated with particle export ratios estimated for the LMEs fishery yields. The *pe*-ratios computed for the LMEs were within the range of 0.20–0.55 and thus well within the model range prescribed by Dunne et al. [22] of 0.04–0.72. The *pe*-ratio was most influenced by variation in LME temperature, with lower export ratios associated with higher temperatures, and to a lesser extent affected by NPP/Z_eu_ (Fig. 2a). The correlations between total yield and *pe*-ratio were highly significant (P<0.01) in both untransformed and transformed data treatments (Figs. 5a&b). All *pe*-ratio-yield summary correlates were highly significant (Table 2). Total yield was also significantly correlated with *pe*-flux using both untransformed and transformed data, albeit at a lower level of association (Figs. 5c&d).

### Association Between *z*-ratio and Mesozooplankton Productivity and Yield

**Figure 6:**
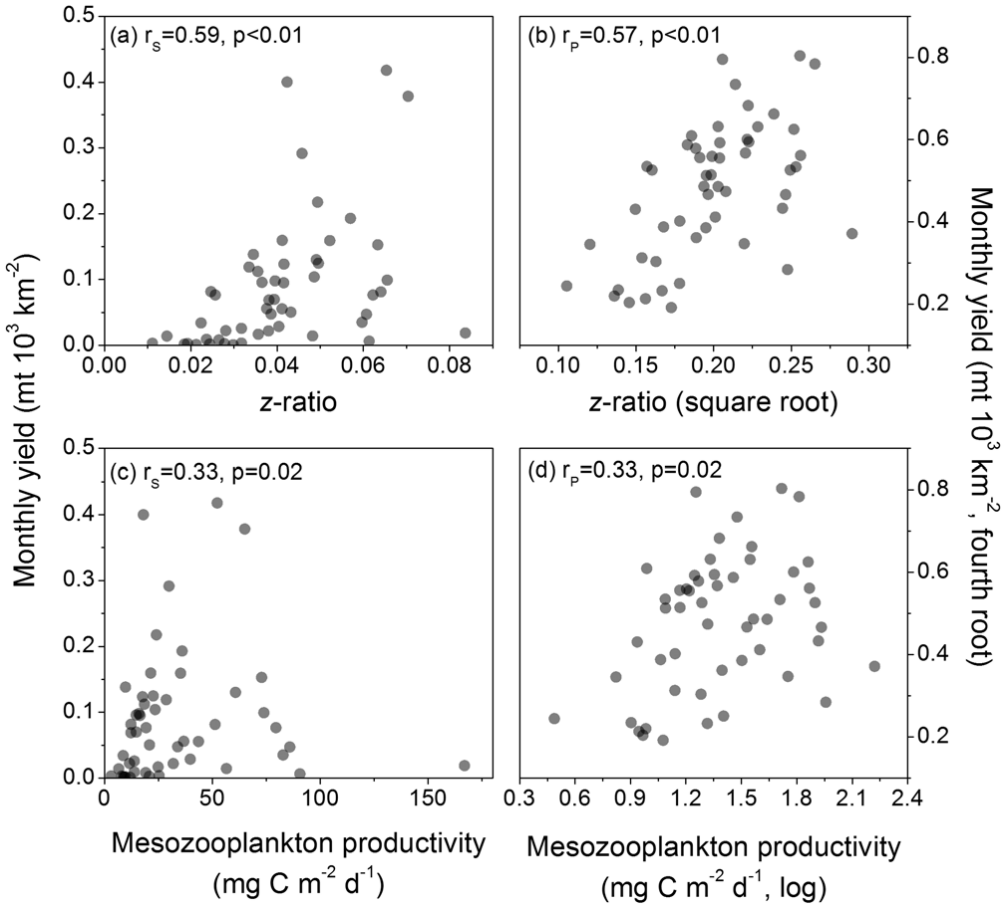
Fishery yield versus *z*-ratio and mesozooplankton productivity. Scattergrams of: (a) total monthly fishery yield and *z*-ratio; (b) same data as panel (a) transformed to approximate bivariate normality; (c) total monthly fishery yield and mesozooplankton productivity computed using the *Eppley*-VGPM algorithm; and (d) same data as panel (c) transformed to approximate bivariate normality. N = 52, the total number of LMEs in the analysis, for all plots. r_S_ = Spearman rank-order correlation and r_P_ = Pearson product-moment correlation.

The *z*-ratios were estimated over a representative range of the model space and like *pe*-ratios were highly correlated with fishery yields. The *z*-ratio was influenced in a balanced fashion by variation in LME temperature and NPP with an increasing trend in *z*-ratio associated with increasing NPP and declining SST (Fig. 2b). The correlations between total yield and *z*-ratio were significant for both untransformed and transformed data treatments (Figs. 6a&b). All the *z*-ratio-yield summary correlates were highly significant (Table 2). Total yield was correlated with mesozooplankton productivity at marginally significant levels (Figs. 6c&d); mesozooplankton productivity-yield summary correlates were marginally significant and in the case of the pelagic summary using transformed data were found to be non-significant (Table 2).

### Multivariate Analysis

For all fishery yield groups, the first principle component explained greater than 40% of the variance in fishery yield (Table 3). In all cases, the second component explained less than 1% of the variance implying a high degree of correlation and collinearity amongst the predictor variables and thus diminishing return from adding new variables; these models were not examined further. The relative correlations between independent variables and principal components closely paralleled the Spearman and Pearson correlation results with chlorophyll *a* and *pe*-ratio having the strongest correlation and NPP the weakest correlation.

**Table 3:** Results from the PLSR analysis with percentage of fishery yield explained by the first component and correlations between independent variables and the first component axis.

	Total	Pelagic	Demersal	Resident
Variance Explained	52.8%	51.6%	41.4%	55.4%
Correlation with independent variables				
Sea Surface Temperature	−0.66	−0.72	−0.56	−0.71
|Latitude|	0.54	0.61	0.44	0.60
Chlorophyll *a*	0.97	0.95	0.98	0.95
Net primary production	0.18	0.08	0.29	0.10
*pe*-ratio	0.92	0.95	0.87	0.95
*pe*-flux	0.63	0.55	0.72	0.57
z-ratio	0.86	0.81	0.91	0.82
Mesozooplankton productivity	0.56	0.48	0.66	0.50

## Discussion

We report significant associations between fisheries yields in 52 globally distributed LMEs and every environmental variable considered herein with the exception of NPP. This result indicates that the relationship between NPP and upper trophic level yield is strongly influenced by factors related to the trophic processes that define the movement of energy to upper trophic levels. Consideration of variations in planktonic foodweb structure associated with changes in NPP and temperature within an ecosystem is essential for robust prediction of fisheries yields across vastly different ecosystems. These conclusion held for both the untransformed and transformed variables.

The variables that did result in a significant linear relationship to fishery yields among the LMEs examined differ in both the correlation strength and significance levels achieved and the clarity of the mechanistic connection associated with them. SST and the absolute value of latitude were moderately correlated to total, pelagic and resident yields and weakly correlated to demersal yields. The ecological mechanisms responsible for these correlations, however, are ambiguous because latitude is a proxy for other factors, including temperature. The linkages between SST and ecosystem dynamics are extremely diverse. For example, SST is linked to the surface ocean stratification which influences the mixing of nutrient rich deep waters to the surface ocean, planktonic productivity, phytoplankton blooms, and phytoplankton community composition [45], [46], [47]. Temperature also exerts direct influence on the vital metabolic rates of organisms within an ecosystem [48].

The observed scaling of fishery yield with temperature and latitude may be the result of the suppressed rates of herbivory by microbial grazers at low temperatures. Such conditions are typical of early spring in temperate latitudes and throughout the year at high latitudes [49]. If a relatively small proportion of primary production goes through the microbial food web under these conditions, the trophic efficiency could be greatly increased as mesozooplankton adapt to consume the smaller phytoplankton normally grazed by microzooplankton (e.g., microphagy). The observation that high latitude systems have more productive fisheries [50], [51], [52] can be partially attributed to the composition of the lower trophic level communities; the productivity of lower trophic levels in high latitudes is thought to be absolutely lower or equal to (but not greater than) those of temperate and tropical systems [13], [18]. Additionally, the maturity (i.e. network properties indicative of flow patterns, complexity and resilience) of high latitude systems and their food web networks are known to be considerably lower than lower latitude systems, with higher energy transfer efficiencies in higher latitude, simpler food webs are a key contributor to this observation [53], [54]. Which combination of these mechanisms contributes to the scaling between temperature and fisheries yields is not clear. Predictors with limited mechanistic underpinning often prove unreliable [55], particularly for applications involving climate variability and change [56].

The concentration of chlorophyll *a* was highly associated with fisheries yield, supporting its utility as a useful indicator of fisheries production at both regional and global scales. However, chlorophyll has been invoked as an indicator of both primary production (a flux) and phytoplankton biomass (a scalar). Both of these interpretations have serious limitations at global scales. The assignment of high chlorophyll concentration in an environment as a proxy for phytoplankton biomass can be problematic. Chlorophyll concentration can indicate the potential of substantial net primary production, but also the capacity of the phytoplankton assemblage to outgrow and/or inhibit predation activity [57]. The occurrence of phytoplankton blooms in the marine environment has long been recognized as a combination of both sustained growth by primary producers and escape from top-down controls on population size by the grazing activities of micro- and mesozooplankton [58]. The existence of a strong relationship between chlorophyll and fisheries yields contrasts with the lack of a relationship between NPP and fisheries yields. Such a confounding relationship illustrates the complexity of the relationship between chlorophyll and NPP. Furthermore, variations in the ratio of phytoplankton chlorophyll to carbon [59], [60] complicate the interpretation of chlorophyll as a measure of phytoplankton biomass.

While the detection of chlorophyll is more directly indicative of the dynamics of the lower trophic level of a region than is latitude or SST, a more complete understanding of the mechanisms underlying the global scale relationship of chlorophyll and fisheries yield is needed. Like chlorophyll *a*, particle export flux and the ratio of mesozooplankton production to primary production are highly correlated with fishery yields on a global basis. The former is directly related to measures of the ratio of new primary production to total production (the sum of new and recycled production). New primary production is directly available to mesozooplankton which in turn supports production of upper trophic levels. The ratio of mesozooplankton production to primary production reflects transfer efficiencies between two critical components of the food web affecting fishery productivity. These metrics provide more detailed insights to fishery production throughout the world ocean than chlorophyll concentration alone. The additional mechanistic detail, coupled with their ability to explore different pathways and respond to more explicitly known processes offsets the less than straightforward calculations needed to obtain such measures as compared to chlorophyll *a* estimates. Although all three measures are associated with global fisheries yields, the rationale for why one would want to use these rate measures beyond the easier to measure and obtain chlorophyll *a* estimates reside in the purpose of exploring such relationships; simple and cursory predictions may imply chlorophyll would be fine in some circumstances, whereas more nuanced considerations and explorations would likely merit use of the rate measures we describe here.

Particle export flux and mesozooplankton production are both estimates of the export of energy from the planktonic foodweb to forms more readily available to fisheries (i.e., large sinking particles and mesozooplankton) and offer deeper mechanistic understanding still of fishery production processes. Such a connection can be used to understand differences in yield among regions. Two major patterns are apparent in the particle flux and mesozooplankton production indices: first , there is a non-linear transition from low to high *pe*- and *z*-ratios with increasing NPP, and second, SST is negatively correlated with both ratios for the vast majority of NPP values. These two patterns correlate to the observed high fishery yields in moderate to highly productive ecosystems in temperate and sub-polar regions, producing improved correlations relative to primary production.

A major driver of the shift from low *pe*-/*z*-ratios and particle export fluxes/mesozooplankton production to high values with increasing NPP (Fig. 2) is the transition of planktonic ecosystems from one dominated by pico-phytoplankton, microzooplankton and the microbial loop to one dominated by large phytoplankton and mesozooplankton [22], [41]. The observed transition in phytoplankton composition among these ecosystem states is consistent with the succession of larger phytoplankton as nutrient supply and productivity increases [61]. Small phytoplankton dominate low nutrient environments because they are superior nutrient scavengers as a result of their relatively high surface area to volume ratio [62]. As nutrients increase, small phytoplankton population growth rates are maximized and population biomass is mediated by microzooplankton grazing. Populations of progressively larger phytoplankton are then established. Larger phytoplankton are consumed by larger predators [63] serving to decrease the number of trophic links between phytoplankton and fish. Fewer and more direct trophic connections lead to the production of larger, faster sinking particles. Increasing zooplankton gross growth efficiency also contributes to an increased *z*-ratio because ingestion rates become large relative to basal metabolic costs in more productive ecosystems. These explanatory mechanisms are consistent with Ryther's theoretical arguments for the relationship between primary production and fish production [18].

Temperature acts on the *pe*- and *z*-ratio in a number of ways to create the negative correlation with SST. First, faster remineralization rates in warmer temperatures decrease *pe*-ratios in warm water ecosystems [21]. Second, increasing water temperature moves the transition from a small to a large phytoplankton dominated ecosystem to higher values of NPP [41]. In the model used to derive *z*-ratio, simultaneous increases in phytoplankton growth rates and zooplankton ingestion rates with increasing temperature stabilizes the biomass at which small phytoplankton growth nears its maximum and large phytoplankton becomes prevalent. The small phytoplankton growth rate at this transition point, however, is higher at higher temperatures [38]. Since NPP is the product of the growth rate and the phytoplankton biomass, the transition point to a large phytoplankton dominated ecosystem moves to higher values of NPP. Assuming differential temperature dependencies between phytoplankton and zooplankton [49], [64] can modulate this pattern, but does not eliminate it. The pattern also emerges from independent *z*-ratio estimates derived from a combination of *in-situ* data and empirical models [41].

There was no strong evidence in the analysis that pelagic and demersal fisheries respond differently to primary production and rates related to trophic transfer. In particular, estimated particle export flux and mesozooplankton production performed similarly well as predictors of both pelagic and demersal fish, though the former flux is thought to primarily fuel benthic production and the later pelagic. This in part reflects the similarity of emergent trends in both quantities that arises for the common linkage of these quantities to changes in planktonic foodweb structure. It may also reflect the prevalence of fish that interact with both the demersal and pelagic foodwebs, supporting the interpretation that many functional groups assumed to feed in a particular sector of the water column likely feed in multiple sectors. As has been noted, many of these fishes show clear feeding tendencies, but even appropriation into feeding guilds still exhibits high dietary overlaps across guilds [65], [66], [67], [68].

There are a number of limitations of the analyses herein that will be addressed in future work. First, the observed patterns in *pe*- and *z*-ratios represent low-frequency broad-scale changes that underlie significant unresolved spatial and temporal variations. In the case of the *z*-ratio, the modeled transition to high *z*-ratio state is delayed relative to independent *z*-ratio estimates. Analysis against region-specific data and further improvement of these models is needed. A second consideration is the potential impact of uncertainties in satellite-based NPP measurements; as model validation improves with improving global calibration data, NPP estimates should be re-evaluated. Thirdly, NPP temporal variability has recently been associated with catch trophic level and yield leading to the suggestion that lower yields are associated with less variable ecosystems and higher yield associated with greater variability [69]; this result would seem to be consistent with the intent of the findings in this paper. But, the same report also suggests that high temporal NPP variability favors demersal production, while pelagic yield is favored by lower temporal NPP variability, a result that might be expected to produce greater separation in yield groupings associated with geographic variation, visualized with proxies like latitude or SST [70], a result not clearly addressed here.

Accounting for variations in the dynamics of planktonic ecosystems in the context of NPP and temperature change remains an essential step for understanding upper trophic level yields. Understanding these trophic linkages should guide more parsimonious ecosystem model development, especially as it relates to linked ecosystem models that may be applied to fishery and marine spatial planning problems. The prominence of the association between yield and *pe*-ratio also suggests that ecosystem models not only need to account for lower trophic level linkages, but should also explicitly model the vertical distribution of organic carbon. It would seem the dimensional distribution of particulate carbon energy may be important to movement of energy up the food chain and that reduction of energy distribution from three to two dimension plays a role. It would be useful to explore other measures of energy movement beyond *pe*-ratio which is tied to rates of primary production. It may be useful to consider rates independent of phytoplankton production or rates related to plankton biomass. Furthermore, it would be useful to explore regional and time series data to see if relationships could be developed to estimate yield potential based on the spatial aspects of the trophic transfer of primary production.
